# Synchronous Nasopharyngeal and Thyroid Malignancies: A Case Report

**DOI:** 10.7759/cureus.41674

**Published:** 2023-07-10

**Authors:** Mohammed A Basyuni, Abdulaziz Altowairqi, Meshal F Khan, Ahmed S Bahaj, Jabir Alharbi, Mohammad A Alessa, Sherif K Abdelmonim

**Affiliations:** 1 ENT Head and Neck Surgery, Otolaryngology-Head and Neck Surgery, King Abdullah Medical City, Makkah, SAU; 2 Otolaryngology-Head and Neck Surgery, King Abdulaziz Specialist Hospital, Taif, SAU; 3 Otolaryngology-Head and Neck Surgery, Al-Noor Specialist Hospital, Makkah, SAU; 4 Otolaryngology-Head and Neck Surgery, King Abdullah Medical City, Makkah, SAU; 5 Otolaryngology, Majmaah University, Majmaah, SAU; 6 Head, Neck and Skull Base Health Center, King Abdullah Medical City, Makkah, SAU; 7 Otolaryngology-Head and Neck Surgery, Ain Shams University, Cairo, EGY

**Keywords:** multidisciplinary approach, treatment, diagnosis, head and neck, synchronous primary malignancies

## Abstract

This report presents a case of primary non-keratinizing, undifferentiated nasopharyngeal cancer and an oncocytic (Hurthle cell) thyroid carcinoma developing simultaneously. The patient was diagnosed in August 2022 with nasopharyngeal carcinoma. After the staging process and before starting treatment for the patient, he was diagnosed with oncocytic (Hurthle cell) thyroid carcinoma on October 2022. Synchronous primary head and neck malignancies are well-known in the medical field. However, this is a rare case of two primary tumors of mucosal and non-mucosal carcinomas, highlighting the importance of discussing head and neck malignant cases in the multidisciplinary team meeting and performing frequent imaging and endoscopic examination for suspicious cases, especially in elderly patients. This case report describes the cases, the management modalities, and the outcomes, informing clinicians of the importance of considering the possibility of multiple primary malignancies when evaluating patients with head and neck tumors and a better approach to this rare and challenging case to ensure successful management.

## Introduction

The second primary tumor is called synchronous when it is found at the same time or within six months of the diagnosis of the primary malignancy, and it is called metachronous when it is found later than six months after the primary malignancy [1]. The incidence of two primary cancers in one patient, whether synchronous (simultaneous) or metachronous, is not uncommon, especially in the head and neck regions [2]. Primary malignancies of the head and neck patients are more likely to develop secondary cancers. According to Schwartz et al. [3], patients with first-head and neck malignancies experienced a 19% incidence of second primary cancer in the head and neck region, of which 41% were synchronous and 59% were metachronous. An increased index of suspicion of multiple primary tumors in the same patient is essential for patients with head and neck neoplasms to allow additional efforts in the workup, treatment planning, and follow-up visits in patients as the development of a second cancer is life-threatening [3,4].

There have been reports of secondary cancers arising from primary head and neck cancers, and their prognosis remains poorer compared to patients with one primary cancer. Research indicated that from the time of the first primary cancer diagnosis, patients with synchronous tumors have a shorter survival period than patients with metachronous tumors (p<0.001) [5]. On the other hand, the prognosis of metachronous tumors is significantly higher than that of synchronous tumors [5]. Synchronous tumors present unique challenges related to diagnosing and staging, sequence in treatment modalities, intricate surgical plans, treatment toxicity intolerance, etc. [6].

Herein, we report a 75-year-old man with primary non-keratinizing, undifferentiated nasopharyngeal cancer and an oncocytic (Hurthle cell) thyroid carcinoma developing simultaneously in the same patient, which is rarely reported. This is one of the rare cases of two primary tumors of mucosal and non-mucosal carcinoma in the head and neck reported in Saudi Arabia.

## Case presentation

**Figure 1:**
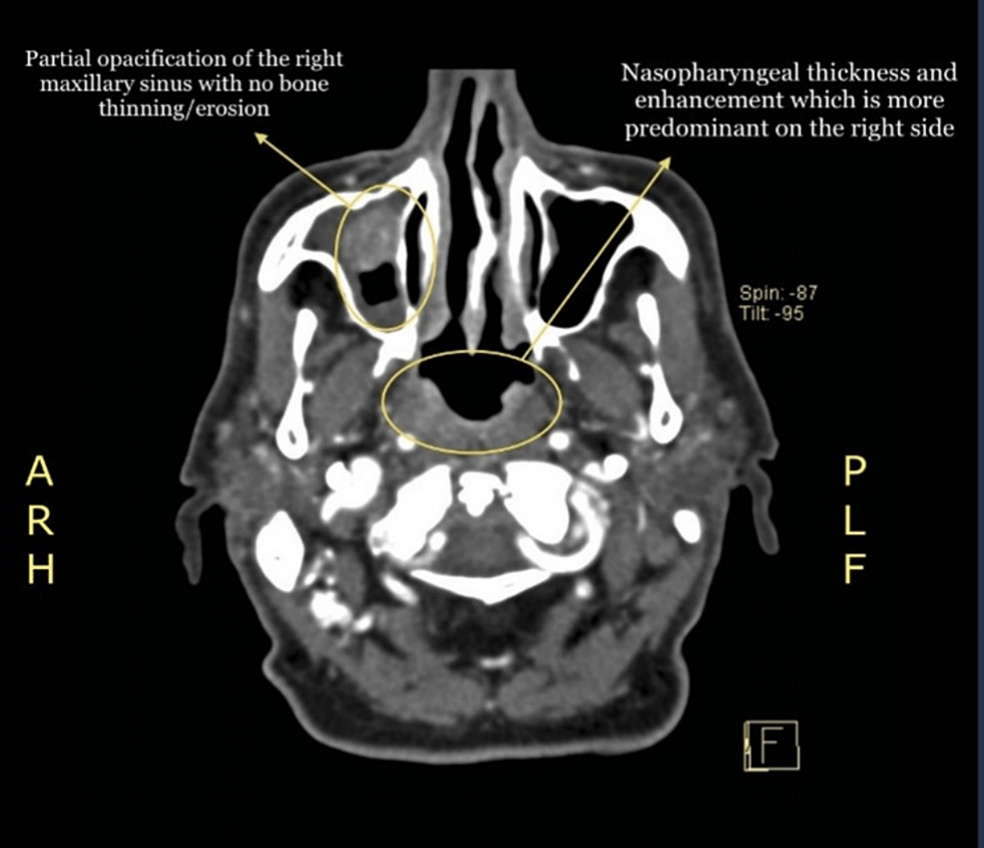
Nasopharyngeal thickening and enhancement.

A 75-year-old hypertensive Saudi man presented on June 2022 to the Otorhinolaryngology Department of King Abdulaziz Specialist Hospital in Taif, Saudi Arabia, with a history of right-sided nasal obstruction and difficulty in swallowing. He had palpable neck swelling, and nasoendoscopy showed a right-sided nasopharyngeal mass whose biopsy test results of August 2022 showed undifferentiated carcinoma. The patient was then referred to King Abdullah Medical City in Makkah for further investigation and treatment, where he underwent a complete staging workup, including head and neck, chest, abdomen, and pelvis computed tomography (CT) that revealed nasopharyngeal thickening and enhancement, predominant on the right side and no enlarged cervical lymph nodes (Figure 1).

**Figure 2:**
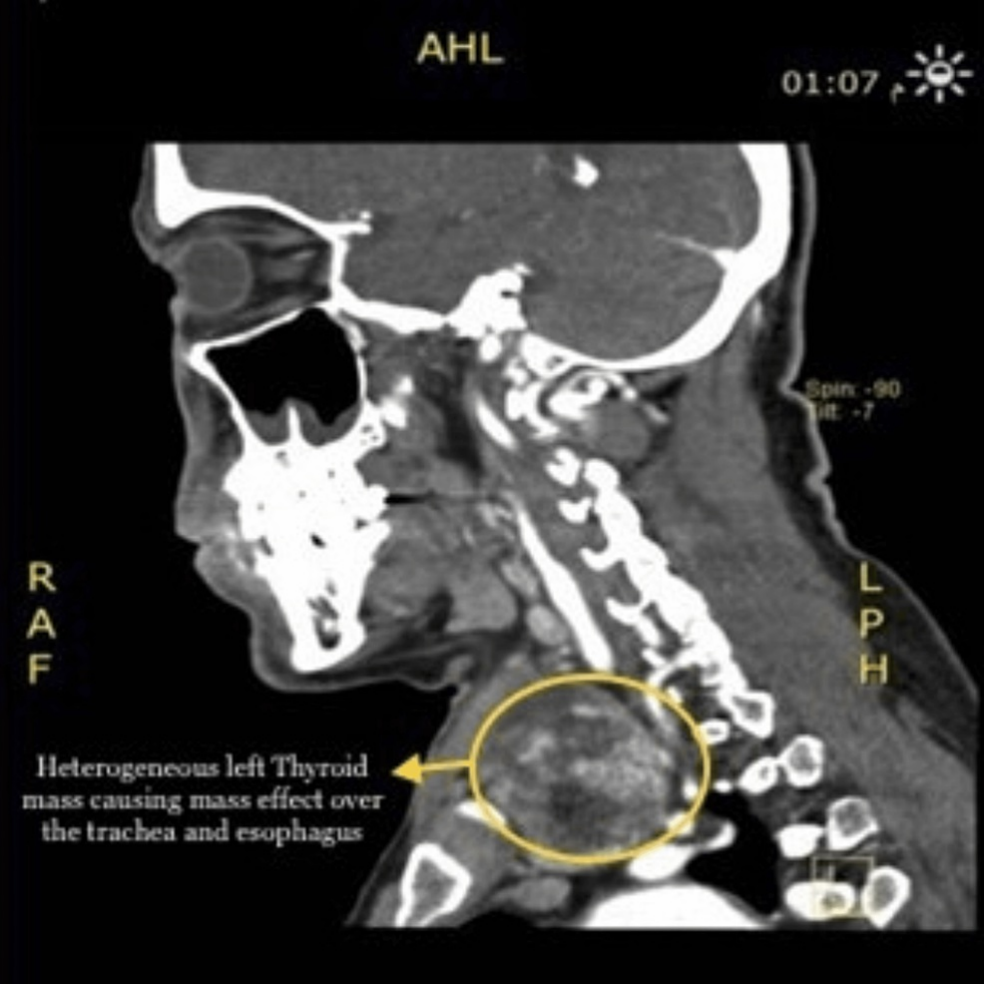
Heterogeneous left thyroid mass causing mass effect over the trachea and esophagus, displacing them to the contralateral side.

**Figure 3:**
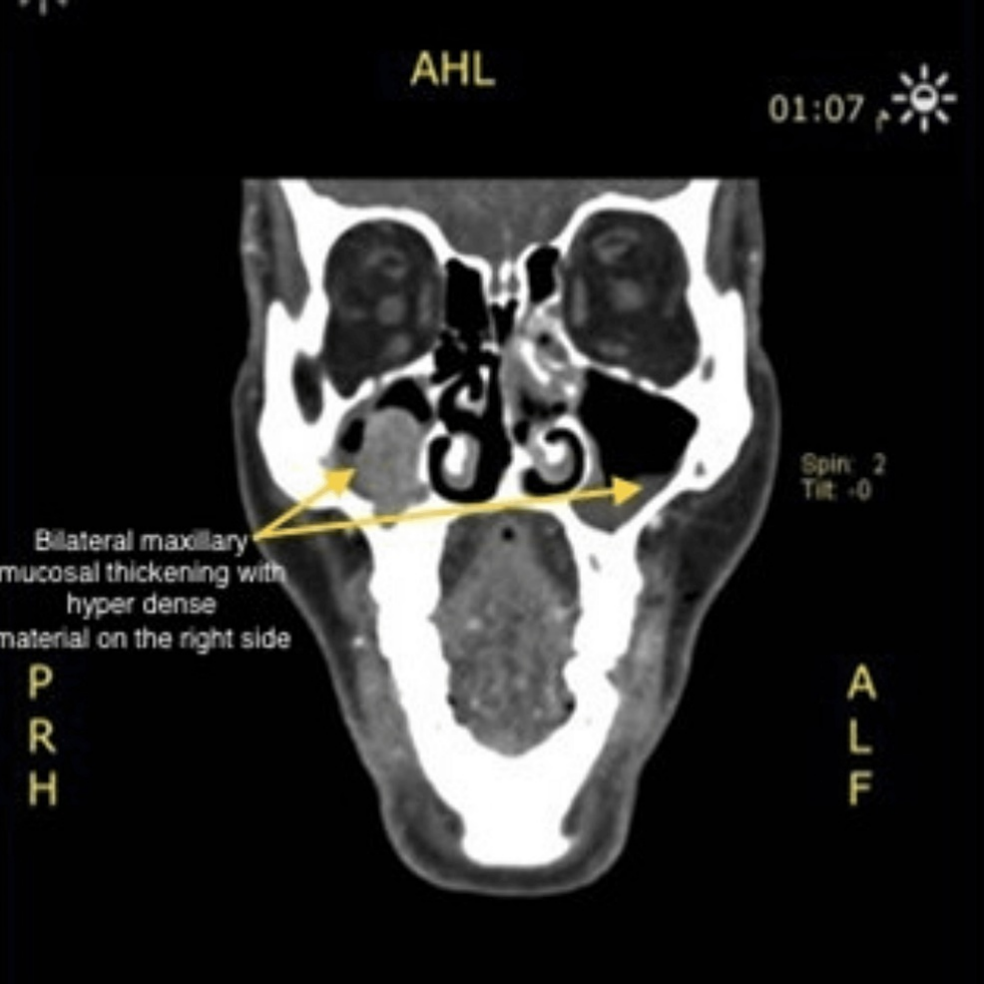
Bilateral maxillary and left ethmoid sinuses mucosal thickening with hyperdense material in the right maxillary sinus.

There was a large well-defined heterogeneous left thyroid mass causing a mass effect over the trachea and esophagus, displacing them to the contralateral side (Figure 2). Mucosal thickening was identified in the bilateral maxillary and left ethmoid sinuses, with hyperdense material in the right maxillary sinus (Figure 3).

Further neck ultrasonography showed similar thyroid gland findings with left moderately suspicious thyroid nodules. Thyroid function tests (TFTs) and serum calcium levels were within normal limits. Fine-needle aspiration cytology (FNAC) of the left thyroid nodules was done and showed benign results. The remaining staging workup did not show distant metastases. A tumor board meeting on September 12, 2022, planned for left hemithyroidectomy because of compressive symptoms, then to be sent to the Oncology Department for chemoradiation for nasopharyngeal carcinoma.

Left hemithyroidectomy was done on October 4, 2022, and the result of histopathology showed oncocytic (Hurthle) carcinoma, minimally invasive, with free margins, intermediate angioinvasion, and without lymphatic or perineural invasion pT3a. The completion thyroidectomy was scheduled twice, but the procedure was canceled because of out-of-hand issues.

**Figure 4:**
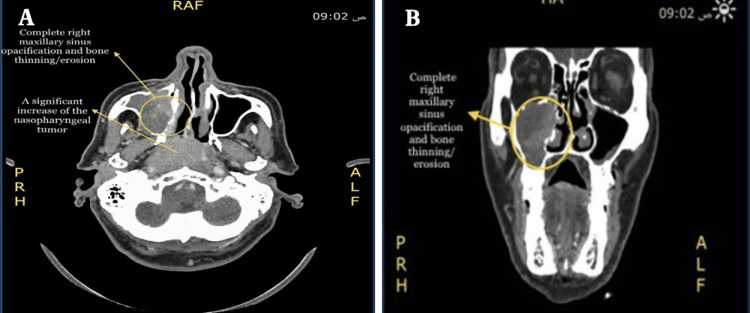
A significant increase in the size of the nasopharyngeal mass (A) and a heterogeneous enhanced soft tissue lesion involving the anteriomedial aspect of the right maxillary sinus with complete opacification of the maxillary sinus and bone thinning/erosion (B).

The new CT scan of the head and neck showed a significant increase in the size of the nasopharyngeal mass, mildly extending anteriorly to the nasal cavity, inferiorly to the oropharynx, right palatine tonsils, and right parapharyngeal, and appearing inseparable from the internal carotid artery below the skull base with no evidence of thrombosis. It also showed the development of large right cervical lymph nodes at levels V, II, and III, with the largest at level V measuring 1.8 × 1.6 cm. There was a significant progression of a heterogeneous enhanced soft tissue lesion involving the anteriomedial aspect of the right maxillary sinus with complete opacification of the maxillary sinus and bone thinning/erosion, and its biopsy showed a primary non-kertinizing undifferentiated nasopharyngeal cancer (Figure 4B). On November 21, 2022, a multidisciplinary team decided to restage the patient as T3N1M0 (Figure 4A) because of the long duration of waiting for the procedure (seven weeks).

**Figure 5:**
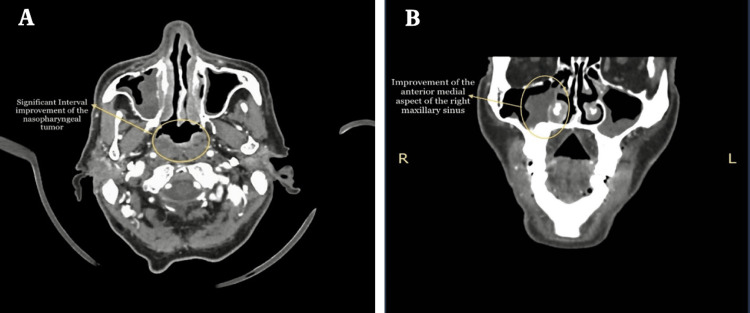
Significant interval improvement of the previously large posterior nasopharyngeal tumor (A), and significant interval improvement of the anterior medial aspect of the right maxillary sinus (B).

The patient was referred to the Radiation Oncology and Medical Oncology departments for concurrent chemoradiation therapy (CCRT) (cisplatin and a total dose of 64 grays for 35 fractions) initiated from November 27, 2022, to January 19, 2023. The CCRT resulted in interval improvement of the previously large posterior nasopharyngeal lesion and an enhancing lesion in the anterior medial aspect of the right maxillary sinus and complete resolution of previously multiple enlarged cervical lymphadenopathies evidenced by CT neck with contrast (Figure 5) and with free CT chest, abdomen, and pelvis. A completion thyroidectomy was done on May 09, 2023 (after 15 weeks after finishing the CCRT), and the patient was scheduled for a follow-up regarding his nasopharyngeal cancer in the combined clinic.

## Discussion

Due to increased awareness of the possibility of a second malignancy, increased use and sensitivity of diagnostic techniques, as well as recent advancements in cancer treatment and survival, the diagnosis of multiple primary tumors is now more common [7,8].

The incidence of multiple tumors occurring simultaneously in different locations within the body was reported to be up to 10% [4]. A meta-analysis showed that the incidence of second, third, and fourth primary tumors is 3-5%, 0.5%, and 0.3%, respectively [4]. Second primary tumors were reported to most commonly occur in the esophagus, hypopharynx, buccal cavity, and lung [9]. Although some pathogenic factors, including persistent carcinogenic influence, field cancerization, rising systemic chemotherapy or radiotherapy use, hormonal manipulation, targeted therapy, genetic alteration, and immune suppression, are implicated, the exact cause of multiple cancers developing at the same time is unknown [4,5]. Panosetti et al. showed that 42% of head and neck primary tumors were synchronous, while 58% were metachronous. Survival rates were dependent on the treatment (five-year survival was 8% with modified treatments, compared to 28% with unmodified treatments [10]. Studies showed that the five-year survival rate was 18% among synchronous cancer patients compared to 41-55% among metachronous cancer patients [7]. Another study found that the five-year survival among patients with second primary cancer was 26% from the time of diagnosis [11]. It was found that the prognosis for synchronous cancers deteriorates if the planned treatment has to be modified following the discovery of a second primary [12]. The tumor-specific mortality for patients with a second primary tumor was found to be 20% after 15 years compared with 44% for patients without a second primary tumor [11].

A thorough family history review and follow-up for the emergence of subsequent primary tumors should be conducted on people with a history of multiple malignancies [7,12]. Cancer screening, genetic counseling, risk assessment, and cancer screening must all be prioritized. Additionally, every new tumor must be biopsied [12].

The second primary tumor is typically more aggressive, treatment-resistant, and metastasizes earlier, necessitating a more aggressive treatment regimen [13]. However, how the initial tumor affects the second main or vice versa is still unclear. Treatment strategies should be directed toward the most advanced or aggressive malignancy for both simultaneously [13,14]. For our patient, we treated the thyroid tumor first as it was compressing the trachea and esophagus, which could result in severe morbidities and higher mortality odds than nasopharyngeal tumors.

For the best chance of finding second primaries in patients with head and neck cancers, performing a routine interval endoscopic intervention within two years of treatment is advised. Additionally, a lifetime clinical surveillance of second neoplasms of the aerodigestive tract in cancer patients with cancers of the oral cavity, oropharynx, and hypopharynx is necessary [15,16]. This is because head and neck second primary tumors are more common in the aerodigestive tract [16]. 18F-fluorodeoxyglucose (FDG) PET/CT is vital for surveillance, detecting recurrence, and finding second primaries throughout the whole body, including the head, neck, and thyroid regions [17].

## Conclusions

Despite the rarity of two main tumors in the head and neck region, early detection of any subsequent primary tumors in patients with head and neck malignancies should get particular attention. Therefore, a multidisciplinary approach involving oncologists, surgeons, radiologists, pathologists, and other healthcare professionals and individualized treatment plans for each patient’s overall health is essential to optimize outcomes.
